# Comprehensive Genome Profiling Test in Japanese Patients With Castration-Resistant Prostate Cancer: A Single-Center Retrospective Study

**DOI:** 10.7759/cureus.77300

**Published:** 2025-01-11

**Authors:** Tomohiro Hori, Hiroaki Iwamoto, Tomoyuki Makino, Renato Naito, Hiroshi Yaegashi, Shohei Kawaguchi, Kazuyoshi Shigehara, Takahiro Nohara, Kouji Izumi, Atsushi Mizokami

**Affiliations:** 1 Department of Integrative Cancer Therapy and Urology, Kanazawa University Graduate School of Medical Science, Kanazawa, JPN

**Keywords:** castration-resistant prostate cancer, cdk12, comprehensive genome profiling, high tumor mutation burden, prostate cancer

## Abstract

Background: Castration-resistant prostate cancer (CRPC) represents a significant difficulty in oncology, with limited treatment options and decreasing survival rates. The comprehensive genomic profiling (CGP) test has appeared as a promising tool for personalizing treatment according to the genetic characteristics of tumors. In Japan, the incidence of prostate cancer (PC) has sharply increased, making it crucial to investigate effective therapies informed by genomic data.

Methods: This study retrospectively analyzed data from 30 patients who underwent the CGP test at Kanazawa University from March 2020 to February 2024. Patient information, including age, clinical stage, and previous treatments, was collected. The CGP tests were conducted on the tumor and blood specimens using FoundationOne® CDx. Survival analysis was conducted using the Kaplan-Meier method, with a significance level set at a *p*-value of <0.05.

Results: Genomic mutations were detected in 27 patients (90%), predominantly TP53 (19 cases) and PTEN mutations (10 cases). Five patients received treatment based on the CGP results but with no significant difference in overall survival (OS) between the treated and untreated groups (*p* = 0.72). Notably, patients with CDK12 mutations demonstrated a significantly shorter OS (*p* = 0.032). Pembrolizumab in cases with high tumor mutation burden exhibited limited efficacy.

Conclusions: The CGP test revealed critical genetic mutations in patients with CRPC and highlighted the poor prognosis associated with CDK12 mutations. The results underscore the necessity for novel therapies tailored to these genetic profiles, emphasizing the role of the CGP in improving treatment personalization.

## Introduction

The International Agency for Research on Cancer 2022 reported prostate cancer (PC) as the second most diagnosed cancer and the fifth leading cause of cancer-related death in men globally [1]. In Japan, PC cases have significantly increased recently, making it the most prevalent type of cancer among men in 2019 [2]. Androgen deprivation therapy (ADT) has long been utilized for metastatic PC. However, many PCs progress to castration-resistant prostate cancer (CRPC) after several years of treatment [3]. The recent development of androgen receptor signal inhibitors has significantly changed the treatment approach for metastatic PC, but some patients, such as those with neuroendocrine PC, continue to demonstrate poor treatment response [4-6]. Hence, developing new treatments for CRPC is an urgent need, and the comprehensive gene panel (CGP) test is gaining attention as a part of this process [7].

The CGP test is an approach to analyzing mutations in multiple genes simultaneously, enabling personalized medicine according to the characteristics of individual tumors. In particular, poly-ADP-ribose polymerase (PARP) inhibitors in the presence of BRCA1/2 mutations, entrectinib/larotrectinib in NTRK fusion cases, and pembrolizumab in the high tumor mutation burden (TMB-high) and microsatellite instability-high (MSI-high) cases are the representative molecular-targeted drugs that were available based on the CGP test results [8-10]. In addition, some genetic mutations detected by the CGP test may provide a starting point for new treatments, including clinical trials. Racial differences in PC incidence and mortality have been reported, and genomic profiling helped determine these differences [11]. However, only a few reports described genomic testing in Japanese patients.

Thus, this study summarizes the CGP test results in Japanese patients with PC and reports the genetic mutations determined through the CGP test, along with the corresponding prognosis according to these mutations.

## Materials and methods

We retrospectively investigated the CGP test in patients with CRPC at Kanazawa University from March 2020 to February 2024. Follow-up for this study was completed on August 12, 2024, with a median follow-up period of 13 months. Data on age, clinical stage, and grade group were obtained from the patient’s medical records during PC diagnosis. The clinical stage was identified based on the 2017 TNM grading system, eighth edition [12]. CHAARTED, LATITUDE, and Canazawa criteria were adapted to assess risk classification [5,6,13,14]. CRPC was defined as a prostate-specific antigen (PSA) level increase of at least 2.0 ng/mL and a 25% elevation from the nadir level, as confirmed by a second PSA test at least four weeks later [15]. Data on age, Eastern Cooperative Oncology Group Performance status, metastasis site, PSA level, time from initial treatment to CGP test, metastasis-directed radiotherapy before CGP test, use of bone-modifying agents, and number of treatment lines before CGP test during the CGP test were also collected. The CGP test was conducted using formalin-fixed paraffin-embedded tumor specimens or blood specimens, with either FoundationOne® CDx, Foundation One® Liquid CDx, or the NCC Oncopanel System. In cases where tissue specimens were of insufficient quality or could not be used for testing (e.g., insufficient quantity or degradation), blood specimens were used as an alternative to ensure that the genomic analysis could proceed. Genomic mutations determined in the CGP tests were collected. Survival analyses were conducted for genomic mutations reported to have a poor prognosis, including CDK12, BRCA1/2, TP53, and PTEN [16-18].

All statistical analyses were conducted with IBM SPSS Statistics for Windows, version 22.0 (released 2013, IBM Corp., Armonk, NY). Overall survival (OS) was the time from initial diagnosis to death and was calculated with the Kaplan-Meier method. The statistical significance in all analyses was set as p-values <0.05. The Ethics Committee of Kanazawa University approved the study (approval no. 2020-286), conducted under the Declaration of Helsinki guidelines.

## Results

The CGP tests were conducted in 30 cases. Table 1 presents patient characteristics during the initial PC diagnosis. The median age was 67 years (range: 50-76) and the median PSA level was 42.3 ng/ml (range: 2.3-4,947). The most common grade group was five, accounting for 18 (60%) cases. In the two patients who did not initially undergo a prostate biopsy, the diagnosis of PC was made clinically due to extremely elevated PSA levels, which were considered diagnostic of the condition. Both patients received hormone therapy as initial treatment based on this clinical diagnosis. However, both subsequently underwent a biopsy after CRPC diagnosis. Fifteen (50%) cases were diagnosed as high risk by the LATITUDE criteria and high volume by CHAARTED criteria. Fourteen (46.7%) cases were diagnosed as high risk by the Canazawa risk classification.

**Table 1:** Patient characteristics during prostate cancer diagnosis PSA: prostate-specific antigen

Characteristics	(n = 30)
Median age, years (range)	67 (50-76)
Race, n (%)	
Japanese	30 (100)
Median PSA, ng/ml (range)	42.3 (2.3-4947)
TNM Classification, n (%)	
T2	8 (26.6)
T3	11 (36.7)
T4	11 (36.7)
N0	10 (33.3)
N1	20 (66.7)
M0	11 (36.7)
M1a	0 (0)
M1b	16 (53.3)
M1c	3 (10)
Grade Group, n (%)	
3	1 (3.3)
4	9 (30)
5	18 (60)
Unknown	2 (6.7)
High risk (LATITUDE), n (%)	15 (50)
High volume (CHAARTED), n (%)	15 (50)
High risk (Canazawa), n (%)	14 (46.7)

**Figure 1:**
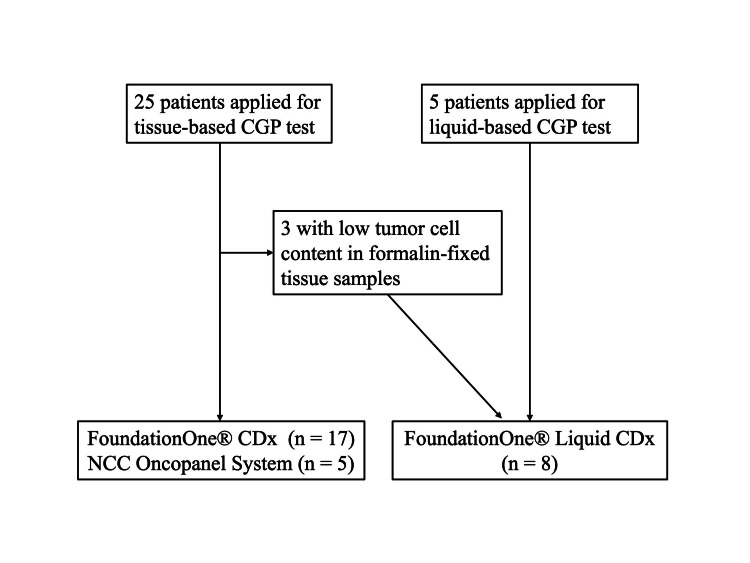
Flowchart of receiving the comprehensive genomic profiling (CGP) test

Table 2 shows patient characteristics during the CGP test. The median age was 72.5 years (range: 52-79) and the median PSA level was 23.1 ng/ml (range 0-389). The median time from initial treatment to CRPC was 19 months (range 3-137) and the median time from initial treatment to CGP was 38.5 months (range 10-187). The median number of treatment lines before the CGP test was 4.5 (range 2-7). FoundationOne® CDx was utilized in 17 (56.6%) cases, FoundationOne® Liquid CDx in eight (26.7%) cases, and NCC Oncopanel in five (16.7%) cases. Figure 1 illustrates a flowchart of the CGP test used in this study.

**Table 2:** Patient characteristics during the CGP test CGP: comprehensive genomic profiling; ECOG PS: Eastern Cooperative Oncology Group performance status; PSA: prostate-specific antigen; CRPC: castration-resistant prostate cancer; MDRT: metastasis-directed radiotherapy; BMA: bone-modifying agents

Characteristics	(n = 30)
Median age, years (range)	72.5 (52-79)
ECOG PS, n (%)	
0	12 (40)
1	18 (60)
Metastasis site at CGP, n (%)	
Lymph node	16 (53.3)
Bone	23 (76.7)
Lung	7 (23.3)
Adrenal gland	3 (10)
Liver	5 (16.7)
Median PSA at CGP, ng/ml (range)	23.1 (0-389)
Median time from initial treatment to CRPC, month (range)	19 (3-137)
Median time from initial treatment to CGP, month (range)	38.5 (10-187)
Median number of treatment lines before CGP (range)	4.5 (2-7)
Type of CGP, n (%)	
FoundationOne® CDx	17 (56.6)
FoundationOne® Liquid CDx	8 (26.7)
OncoGuide™ NCC Oncopanel System	5 (16.7)
Specimen of CGP, n (%)	
Tissue of primary site	17 (56.6)
Tissue of metastatic site	5 (16.7)
Blood	8 (26.7)
MDRT before CGP, n (%)	15 (50)
Use of BMA, n (%)	
Denosumab	13 (43.3)
Zoledronic acid	8 (26.7)

**Figure 2:**
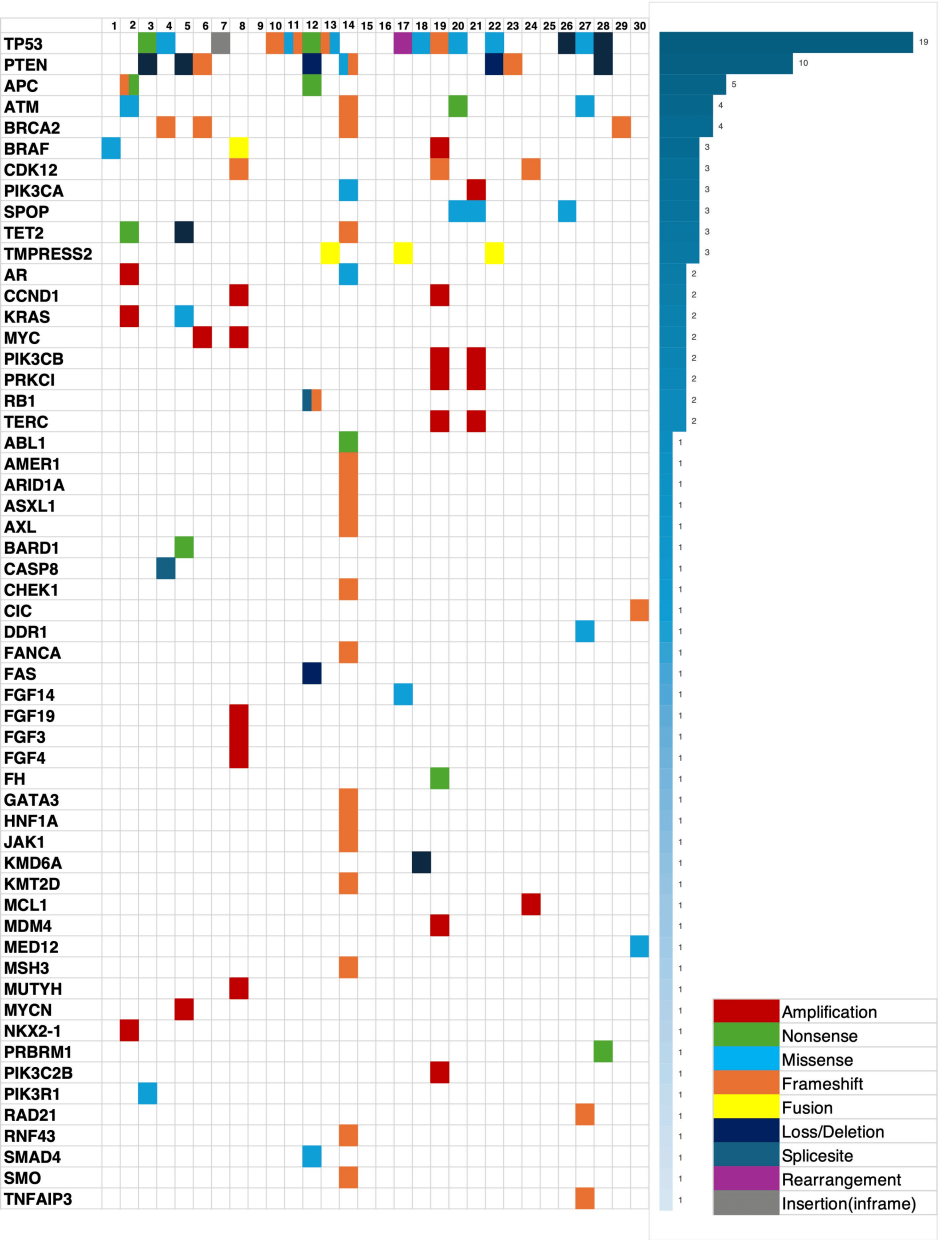
Genomic mutations detected in the comprehensive genomic profiling (CGP) test

Figure 2 summarizes genomic alterations detected in the CGP test. Genomic alterations were observed in 27 out of 30 cases, accounting for 90% of the total cases. Of the genomic alterations, TP53 mutations were the most prevalent (19 cases), followed by PTEN mutations (10 cases). BRCA1/2 mutations were detected in four cases, a TMB ≥10 Muts/Mb was detected in four cases, and MSI-high was detected in one case. In total, 11 patients underwent treatment based on the CGP test. Of these patients, five received the treatments recommended in the CGP test (Table 3). One patient received platinum-based chemotherapy, three received pembrolizumab, and three received olaparib. In two of the five patients, both pembrolizumab and olaparib were available. Three patients demonstrated a PSA 50 response (PSA decline of 50% or more from baseline) after treatment. Two patients were confirmed to have a radiological partial response following the Response Evaluation Criteria in Solid Tumors 1.1. However, six patients who received treatments recommended in the CGP test were unable to receive them because they either did not wish to receive these treatments or their condition deteriorated rapidly after the CGP test.

**Table 3:** Details of patients receiving treatment based on the CGP test TMB: tumor mutation burden; MSI: microsatellite instability; CBZ: cabazitaxel; Abi: abiraterone; Flu: flutamide; GCarbo: gemcitabine-carboplatin; Ola: olaparib; Pem: pembrolizumab; EP: etoposide-cisplatin; DTX: docetaxel; PD: progressive disease; PR: partial response; SD: stable disease

Case	Age at CGP	Target gene	Number of treatment lines before CGP	Treatment after CGP	Length of recommended treatment, months	PSA ≧ 50% response	Radiological response	Status
1	76	CDK12	3	CBZ→Abi→Ethinylestradiol→Flu→GCarbo	GCarbo:1	No data	PD	Dead
2	73	BRCA2 TMB-high	3	CBZ→Ola→Pem	Ola: 12 Pem: 1	Ola:+ Pem:-	Ola:PR Pem:PD	Dead
3	67	BRCA2 TMB-high MSI-high	2	Pem→Ola	Pem:1 Ola:7	Pem:- Ola:+	Pem:PD Ola:PR	Alive
4	77	TMB-high	5	DTX→Pem	Pem:1 (under treatment)	No data	No data	Alive
5	61	BRCA2	2	Ola	Ola:7 (under treatment)	+	SD	Alive

**Figure 3:**
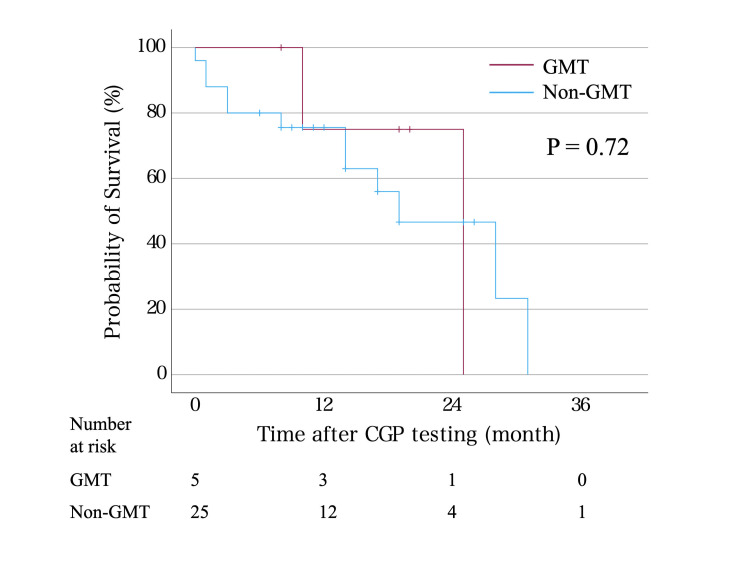
Overall survival after the comprehensive genomic profiling (CGP) test in patients with genotype-matched therapy and others GMT: genotype-matched therapy

Survival analysis was conducted by comparing five patients who received the treatment recommended by the CGP test, with 25 patients who did not receive the recommended treatment (Figure 3). OS after CGP testing indicated no difference between the two groups (p = 0.72).

**Figure 4:**
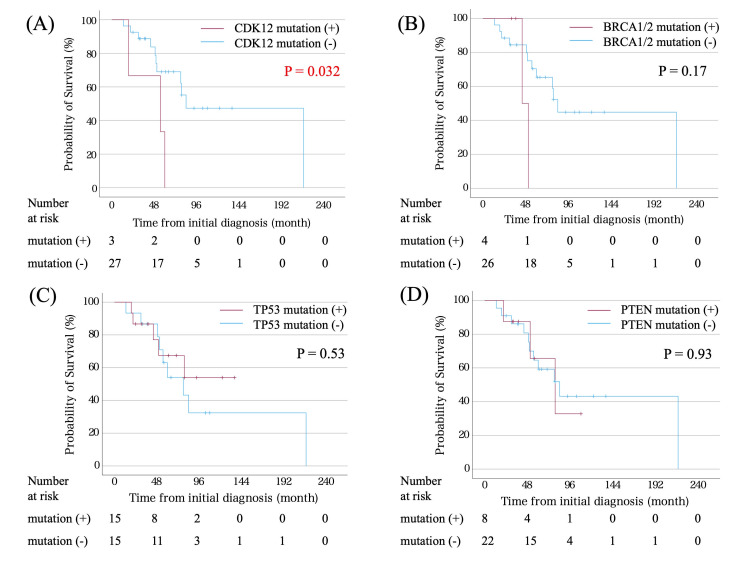
(A) Overall survival in patients with the CDK12, (B) BRCA1/2, (C) TP53, and (D) PTEN mutations compared with others

OS was compared by the presence or absence of genomic alterations detected by the CGP test (Figure 4). OS was significantly shorter in cases with CDK12 gene mutations than in those without mutations (p = 0.032). Conversely, no difference in OS was found for BRCA1/2, PTEN, and TP53 mutations between cases with and without mutations (p = 0.17, 0.93, and 0.53, respectively).

## Discussion

This study presents the genomic mutations determined through the CGP test at Kanazawa University and investigates the differences in prognosis based on the presence or absence of these mutations. The present study indicated that five (16.7%) cases could be treated based on the CGP test results, a proportion generally similar to previous reports published in Japan [19]. To ensure clarity and a comprehensive understanding, we have organized the discussion to focus on each genetic mutation individually.

We revealed significantly shorter OS in cases with CDK12 mutations. CDK12 is responsible for encoding a cyclin-dependent serine/threonine kinase that plays a role in cell cycle and DNA repair regulation through homologous recombination (HR). Previous studies have demonstrated that the loss of function of CDK12 suppresses several HR gene expressions, partly through intronic polyadenylation [20]. CDK12 mutations are observed in 7% of PC cases and are more prevalent in metastatic CRPC (mCRPC) [21]. The present study detected CDK12 mutations in three (10%) cases, a similar rate to previous reports. Patients with CDK12 mutations have had clinical features associated with poor prognosis, including a higher novo metastatic disease prevalence, higher PSA at diagnosis, and a higher Gleason score eight predominance. Patients with the CDK12 mutation have demonstrated a shorter time from ADT introduction to castration resistance and OS from metastatic disease diagnosis [16]. The present study revealed significantly shorter OS in patients with CDK12 mutations (median 55 vs. 84 months, p = 0.032). Previous studies indicated that patients with mCRPC having mutations in DNA damage repair genes, including CDK12, exhibited a better response to platinum-based chemotherapy [22]. These results guided our tumor board to recommend platinum-based chemotherapy for patients with the CDK12 mutation. However, the efficacy of platinum-based chemotherapy may be limited, as in a previous study, wherein four patients with CDK12 mutations were treated with platinum-based chemotherapy and only two of four patients exhibited a PSA 50 response. Patients with CDK12-mutated tumors are suggested to be immunogenic and may benefit from immune checkpoint inhibitors (ICIs), but recent phase Ⅱ trial results have concluded the poor efficacy of ICI therapy in patients with CDK12-mutated mCRPC. CDK12-mutated tumors exhibited a poor prognosis, thereby demanding the development of new therapies in the future.

A meta-analysis reported that 5.26% and 11.26% of patients with mCRPC carried germline and somatic BRCA1/2 mutations, respectively [23]. The present study revealed that four (13.3%) patients had BRCA1/2 mutations, which was generally similar to previous reports. Patients with BRCA1/2 mutations, especially BRCA2, demonstrated significantly worse progression-free survival, OS, and PSA response rates when treated with androgen receptor pathway inhibitors [17]. This study revealed no significant difference in OS between those with and without BRCA1/2 mutations. However, the sample size was small, and further accumulation of cases is warranted.

Pembrolizumab has been effective in patients with mismatch repair gene defects regardless of the primary organ, and TMB-high is related to objective response rates to pembrolizumab in patients with previously treated unresectable or metastatic solid tumors [24,25]. Based on these results, pembrolizumab was approved in Japan for treating MSI-high in December 2018 and TMB-high in February 2022 [26]. However, a recent study revealed no difference in OS between TMB-high and other cases in some cancer types [27]. In addition, the objective response rate in TMB-high PC is lower than that in other cases [28]. The Mayo Clinic has reported that the therapeutic effect of pembrolizumab on mCRPC varies based on TMB levels. The median progression-free survival for TMBs of 10-14.9 mut/Mb, 15-v24.9 mut/Mb, and ≧25 mut/Mb was 2.1 months, not reached (NR), and NR, respectively. The OS for these same groups was 5.1, 20.5, and NR, respectively. However, patients with TMB-high without cooccurring MSI-high or CDK12 mutations experienced no response [29]. Another report compared patients with mCRPC treated with ICI or taxane chemotherapy based on their TMB levels. They revealed that patients with TMB of ≥10 mut/Mb demonstrated a significantly longer time to the next treatment and OS when treated with ICI compared to taxane. However, the cohort of this study demonstrated that most patients with TMB of ≥10 mut/Mb who received ICI also had MSI-high [30]. These studies indicate that pembrolizumab may be less effective in patients with mCRPC with TMB-high mutations without cooccurring MSI-high or CDK12 mutations. Cases of simultaneous BRCA1/2 mutation and TMB-high were found in this study. Clear consensus on whether pembrolizumab or a PARP inhibitor should be administered first in such cases remains unavailable [26]. However, the effect of pembrolizumab on TMB-high is poor in TMB-high cases without cooccurring MSI-high or CDK 12 mutations; thus, treatment targeting those genes may be prioritized if there are other druggable gene mutations.

This study has several limitations. This was a single-center, retrospective study, indicating that clinically important genetic variants may have been missed. This study also lacks a control group, which limits the ability to make direct comparisons between the outcomes of treated and untreated patients or between different treatment strategies. Furthermore, the NCC Oncopanel was utilized as the CGP test in five cases, which may have caused fewer gene mutations to be detected. This limitation could have influenced the comprehensiveness of the genomic data, potentially leading to the underrepresentation of mutations that may have been detected by more comprehensive panels.

## Conclusions

This study indicates a poor prognosis in patients with CDK12 mutations. It also indicates the limited effect of pembrolizumab in patients with TMB-high. These findings emphasize the need for personalized treatment strategies based on genomic profiling, although larger studies are needed to confirm these results and optimize therapeutic approaches.
